# Effectiveness of Telemedicine in Managing Health-Related Issues in the Pediatric Population: A Systematic Review

**DOI:** 10.7759/cureus.72144

**Published:** 2024-10-22

**Authors:** Iman Mohammed Taher Do Alfuqhar, Alaa Eldirdiri Ali Khalafalla, Salma Hassan Mahmoud Ali, Elwaleed Idrees Aydaross Adam, Hanady M Osman, Rwabi Safar Alrabie

**Affiliations:** 1 General Practice, Primary Health Care Center, Emirates Health Services (EHS), Dubai, ARE; 2 Pediatrics, Hull University Teaching Hospitals NHS Trust, Hull Royal Infirmary, Hull, GBR; 3 Medicine, Najran Armed Forces Hospital, Najran, SAU; 4 Pediatrics, Ibri Regional Hospital, Oman, OMN; 5 Family Medicine, Najran Armed Forces Hospital, Najran, SAU

**Keywords:** mobile health technology, pediatrics, sars-cov-2, tele-health, telemedicine

## Abstract

Healthcare delivery is made more convenient and effective via telemedicine, which enables physicians to conduct virtual consultations and evaluations with pediatric patients.

The purpose of this systematic review was to evaluate the efficacy of telemedicine as compared to physical appointments in the pediatric population.

We used Preferred Reporting Items for Systematic Reviews and Meta-Analyses (PRISMA) guidelines to search for the available literature using pre-specified inclusion and exclusion criteria. These databases provided 968 relevant research articles, which Endnote software screened for duplicates. Fourteen studies were considered relevant for full-text evaluation. After complete text evaluation, only 11 of these articles were found to be relevant. The Newcastle-Ottawa Scale (NOS) was used for the risk of bias assessment of all included studies.

Eleven articles in all satisfied the requirements for inclusion and were added to the review. Every study was classified as either a cluster randomized trial (27%) or a randomized controlled trial (RCT) (73%). There were between 22 and 400 participants in each trial. Medical conditions evaluated included obesity (27%), mental health disorders (9%), asthma (18%), otitis media (9%), skin disorders (9%), type 1 diabetes (9%), attention deficit hyperactivity disorder (ADHD) (9%), and pancreatic insufficiency associated with cystic fibrosis (1/11). Telemedicine strategies employed included telemedicine-based screening visits (9%), smartphone-based therapies (27%), phone counseling (18%), and videoconferencing visits between patients and doctors (45%).

The outcomes of the telemedicine procedures in every included study were on par with or superior to those of the control groups. Medication adherence, appointment completion rates, life satisfaction, symptom management, and disease progression were all outcomes associated with these findings. Although more research is needed, the evidence from this review suggests that telemedicine services for the general public and pediatric care are comparable to or better than in-person services. Patients, healthcare professionals, and caregivers may benefit from using both telemedicine services and traditional in-person healthcare services. To maximize the potential of telemedicine, future research should focus on improving patients’ access to care, increasing the cost-effectiveness of telemedicine services, and eliminating barriers to telemedicine use.

## Introduction and background

Telemedicine refers to the use of electronic communications to transfer medical information across locations to enhance a patient's clinical state of health [1]. In the past, the term 'telehealth' has been used more broadly to include catastrophic response, tele-education, tele-research, and clinical patient care provided by telemedicine. In today's context, telemedicine and telehealth are synonymous terms [2].

Since the start of the SARS-CoV-2 pandemic in 2019, telemedicine has been implemented and used widely [3]. Telemedicine is a type of healthcare service where a patient and a distant doctor exchange information using communication technologies. Pediatric patients no longer need to attend their doctors' offices in person for consultations and examinations because telemedicine also makes healthcare delivery more convenient and effective [4].

Pediatric populations experience various health problems that require proper treatment and ongoing management. Chronic illnesses, including asthma, diabetes, mental illnesses, and common developmental issues, cannot be treated in a single visit and require ongoing treatments [5, 6]. Telemedicine has special utility in such situations as it can provide remote consultation and monitoring and ensure timely interventions, all of which should help improve the outcomes of caretaking in children [7]. The utility of telemedicine concerning pediatric health remains less clear, including concerns about how beneficial the technology is as a tool for addressing concerns within this patient population [8].

According to a few studies in the adult population, telemedicine may result in decreased hospitalizations, ER visits, and health-related problems among the general population [9-12]. However, a few Cochrane reviews revealed that there was little evidence to support the idea that telemedicine can enhance outcomes in the pediatric population [6, 13, 14]. In addition, little is known about the effectiveness of telemedicine for pediatric patients. The purpose of this systematic review is to summarize the available literature to determine the effectiveness of telemedicine in preventing and treating various health-related problems among the pediatric population. This review will highlight related research limitations and suggest future research directions to enhance the use of telemedicine in a pediatric setting.

## Review

Methodology

This systematic review of available studies on our topic was conducted according to the PRISMA guidelines ("Preferred Reporting Items for Systematic Reviews and Meta-Analyses") [15].

Search Strategy

We used five different databases to search for published studies in English without restriction on the publishing timeframe. We also checked these databases for the presence of previous or ongoing systematic reviews on the subject. We combined results from different databases and discarded repeated results using Endnote software. The search strategy for each database is presented in Table 1.

**Table 1:** Search strategy for five different databases.

Name of Database	Search Strategy
Scopus	(Telemedicine OR Telehealth OR "Remote Consultation" OR "Virtual Care" OR "Digital Health") AND (Pediatric OR Child OR Adolescent) AND (Effectiveness OR Outcome OR Impact OR "Health-related issues" OR Disease OR Disorder OR Management)
Web of Science	(Telemedicine OR Telehealth OR "Remote Consultation" OR "Virtual Care" OR "Digital Health") AND (Pediatric OR Child OR Adolescen) AND (Effectiveness OR Outcome OR Impact OR "Health-related issues" OR Disease OR Disorder OR Management)
PubMed/EMBASE	((Telemedicine[MeSH] OR Telehealth OR "Remote Consultation" OR "Virtual Care" OR "Digital Health") AND (Pediatric [MeSH] OR Child OR Adolescent)) AND (Effectiveness OR Outcome OR Impact OR "Health-related issues" OR Disease OR Disorder OR Management)
Google Scholar	(Telemedicine OR Telehealth OR "Remote Consultation" OR "Virtual Care" OR "Digital Health") AND (Pediatric OR Child OR Adolescent) AND (Effectiveness OR Outcome OR Impact OR "Health-related issues" OR Disease OR Disorder OR Management)
Cochrane Library	(Telemedicine OR Telehealth OR "Remote Consultation" OR "Virtual Care" OR "Digital Health") AND (Pediatric OR Child OR Adolescent) AND (Effectiveness OR Outcome OR Impact OR "Health-related issues" OR Disease OR Disorder OR Management)

Studies Selection

Each article was retrieved, all duplicates were removed, and each was retained in a separate Endnote library (ENDNOTE, 2015). Two independent reviewers determined which studies should be included. Reviewer 1 (Mahmoud Ali SH) assessed abstracts and titles twice, independently, while Reviewer 2 (Alrabie RS) resolved any disagreements on included studies and approved studies based on the data. Using the inclusion and exclusion criteria listed in Table 2, reviewers carefully assessed the studies to see if they contained the required information for the systematic review.

**Table 2:** Inclusion and exclusion criteria.

Question elements	Inclusion Criteria	Exclusion Criteria
Study type	Quantitative studies, cohort studies, case-control studies, cross-sectional studies, and qualitative studies.	Opinion pieces, editorials, letters to the editor, book reviews, or purely theoretical papers without empirical data.
Population	Studies assessing telemedicine, telehealth, or remote healthcare services used to manage health-related issues.	Studies involving only adult populations or those including adults without a separate analysis for pediatric populations.
Activity	Studies reporting on the effectiveness of telemedicine in improving health outcomes, management of diseases, patient satisfaction, and healthcare access.	Studies that do not assess telemedicine or focus solely on in-person or traditional healthcare methods.
Study Language	Studies published in the English language.	Studies not published in English.

A Microsoft® Excel Spreadsheet was used to extract and store data and records (Microsoft, Inc., Redmond, Washington, USA).

Risk Bias Assessment

The risk of bias in the included studies was evaluated using the Newcastle-Ottawa Scale (NOS). Studies received low, medium, and high scores based on bias in the selection process, interventions, deviations from interventions, missing data, outcomes, and results. Preference for selection was scored using inclusion and exclusion criteria. Performance bias was evaluated by describing a control arm and considering allocation concealment. Complete industry sponsorship, data management, biased reporting, and selective reporting received different rankings. Reviewers (Mahmoud Ali SH and Alrabie RS) reviewed reporting uniformity and eligibility restrictions at multiple meetings. A second reviewer (Alrabie RS) considered any gaps in the reviewers' scores before selecting a study.

Results

Search Results

**Figure 1:**
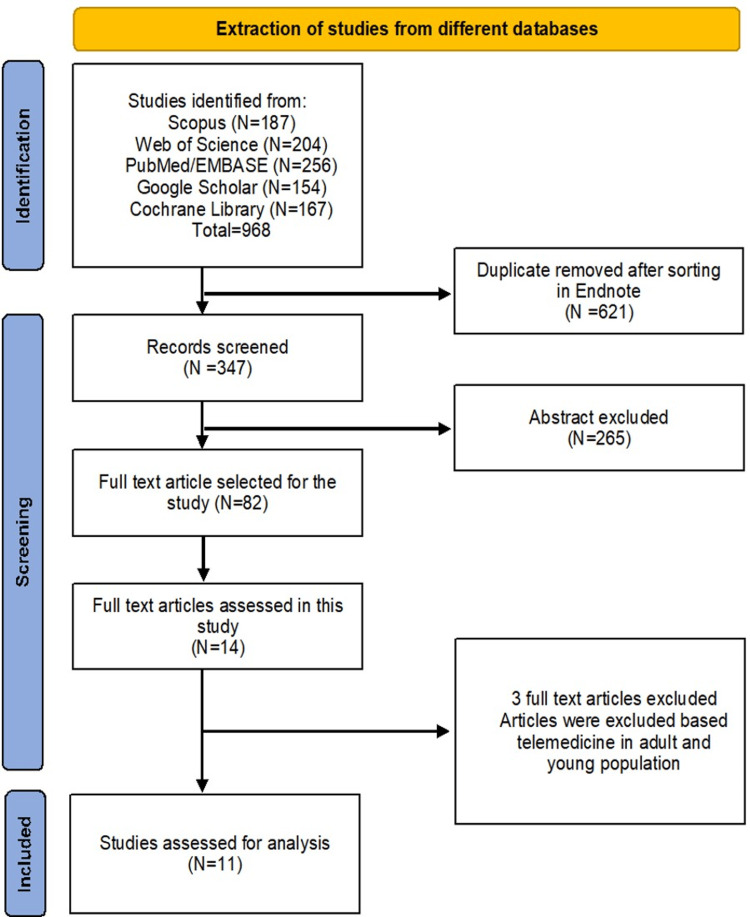
PRISMA flow chart for studies selection. N represents the total number of articles. PRISMA: Preferred Reporting Items for Systematic Reviews and Meta-Analyses.

A total of 968 studies were found across five different databases. When sorted in Endnote, 621 studies were excluded as duplicates. Of the 347 studies screened based on titles and accessibility, 265 were excluded because only the abstract was accessible. Eighty-two full-article texts were retrieved and assessed for eligibility, of which only 14 studies were found to be eligible for our review. After careful examination, three articles were excluded because they focused on telemedicine in adult and young populations. This left 11 studies, which were included in this systematic review (Figure 1).

Risk Bias Assessment

Table 3 shows the risk of bias assessment using the NOS. Out of 11 studies, four showed low risk bias, two showed high risk bias, and the rest exhibited moderate risk bias. In some studies, a portion of their methodological flaw is the way they chose their controls. Furthermore, the inclusion of the review may lead to a moderate risk bias assessment overall.

**Table 3:** Risk of bias assessment in the studies included in this systematic review. Rating scale: 7 to 9 stars = low risk of bias; 4 to 6 stars = moderate risk of bias; 0 to 3 stars = high risk of bias. *Selection:* 1. Is the case definition adequate? 2. Representativeness of cases; 3. Selection of controls (community or hospital); 4. Definition of controls. *Comparability:* 1. Comparability of cases and controls based on the design or analysis. *Exposure:* 1. Determination of exposure; 2. The same calculation method for cases and controls; 3. Non-response rate. A study can receive a maximum of one star (★) for each numbered item in the Selection and Exposure categories. A maximum of two stars (★★) can be given for Comparability.

Study	Selection				Comparability	Exposure		
	1.	2.	3.	4.	1.	1.	2.	3.
Powers SW et al. [16]	★	★			★★	★	★	★
Davis AM et al. [17]	★	★			★	★	★	
Vander SA et al. [18]	★	★				★	★	★
Rhodes ET et al. [19]	★	★						★
Fleischman A et al. [20]	★	★			★★	★	★	★
Di Bartolo P et al. [21]	★	★			★	★	★	★
O'Connor DM et al. [22]	★	★			★★		★	★
Halterman JS et al. [23]	★	★		★	★★	★	★	★
Perry TT et al. [24]	★	★	★		★		★	★
Erkkola-Anttinen N et al. [25]	★	★			★★	★	★	★
Coker TR et al. [26]	★	★					★	

The studies included in this systematic review had a moderate-quality risk bias, according to GRADEpro GDT. The primary reasons for the moderate risk bias assessment were the inclusion of a cohort study, which raises the possibility of bias as it cannot randomize the exposure, and the uneven characteristics of the study.

Characteristics of Included Studies

Table 4 shows the characteristics of each included study. The studies covered numerous medical conditions, including otitis media (1/11, 9%) [25], type 1 diabetes (1/11, 9%) [21], skin conditions (1/11, 9%) [22], hyperactivity disorder (1/11, 9%) [18], asthma (2/11, 18%) [23, 24], obesity (3/11, 27%) [17, 19, 20], mental health conditions (1/11, 9%) [26], and pancreatic insufficiency (1/11, 9%) [16]. Of the eleven included studies, 82% were conducted in the USA, one (9%) in Italy [21], and one (9%) in Finland [25]. All studies were published in English. The sample sizes ranged from 22 to 400 participants. Four studies (36%) [19, 20, 22, 25] had relatively small sample sizes (less than 50 participants), and four more studies (36%) had overall sample sizes of more than 200 participants. Participants' ages ranged from 17.7 years to 21 months on average or median. Of the eleven studies, two (18%) [20, 21] stated that the mean age of individuals was greater than 13 years, while one (9%) stated that the mean age was less than 3 years. Male participants outnumbered female participants in the majority of the trials (7/11, 64%). According to the selection criteria of the present review, all study designs were categorized as either cluster randomized controlled trials (RCT) (3/11, 27%) [17, 24, 26] or RCT (8/11, 73%). The duration of the follow-ups varied from sixty days to five years. Eight (73%) of the eleven included studies had follow-up periods ranging from six to twelve months, whereas one (9%) did not follow up with participants. The majority of primary outcomes focused on patients' symptom changes (8/11, 72%), telemedicine's time efficiency (1/11, 9%), or the agreement between telemedicine and in-person diagnosis (2/11, 18%).

**Table 4:** Characteristics of included studies. ^a^USA: United State of America; ^b^RCT: Randomized Control Trials; ^c^Z-score= Describes a value's relationship to the mean of a group of values; ^d^BMI.

Citation	Publishing Year	Country of Study	Study Design	Sample Size	Mean Age of participants	Health condition	Percentage of Female population	Approach used	Measures Outcome	Follow-up Duration
Powers SW et al. [16]	2015	USA^a^	RCT^b^	78	3.8 years	Pancreatic insufficiency and cystic fibrosis	43%	A portion of each therapy was given over the phone.	Primary: modifications to energy intake; secondary: adjustments to height and weight ^c^Z scores	18 months
Davis AM et al. [17]	2016	USA	Cluster RCT	103	9.14 years	Obesity	55.43%	Families received behavioral group therapies from physicians using telemedicine.	Primary: Z-score for ^d^BMI Secondary: accelerometer data, behavioral checklist results, feeding assessment scale results, 24-hour dietary recall, parents' BMIs, and feasibility measurements	08 months
Vander SA et al. [18]	2017	USA	RCT	223	9.23 years	Hyper activity disorder	29.9%	Video counseling for Telepsychiatry sessions.	Primary: shifts in distress as determined by various surveys Secondary: family empowerment, parenting stress, caregiver stress, and patient health	25 weeks
Rhodes ET et al. [19]	2017	USA	RCT	22	8.1 years	Obesity	54.5%	Dietary sessions via video call.	Primary: changes in glycemic load and total number of calories in fat Secondary: total energy intake	01 year
Fleischman A et al. [20]	2016	USA	RCT	73	14.3 years	Obesity	77.5%	Teleconsultations between doctors and specialists and televisits with obesity specialists.	Primary: BMI changes Secondary: blood pressure, dietary glycemic load, triceps skinfold, waist size, and physical activity	01 year
Di Bartolo P et al. [21]	2017	Italy	RCT	182	17.7 years	Diabetes	48.9%	Health care personnel can receive information straight from glucose meters that link with a phone app. Patients had three options for communicating with doctors: phone, SMS, or email.	Primary: variations in levels of hemoglobin A1c Secondary: the quantity of patients who kept track of their own blood sugar levels and their overall well-being	01 year
O'Connor DM et al. [22]	2017	USA	RCT	40	6.96 years	Skin condition	55%	For direct patient-to-doctor telemedicine, parents took pictures of their child's skin condition using a smartphone.	Primary: Congruence between diagnosis based on photographs and in-person Secondary: the impact of photo instructions, image quality, and parental willingness	Not reported
Halterman JS et al. [23]	2018	USA	RCT	400	7.8 years	Asthma	38.25%	Telemedicine visits in schools	Primary: the quantity of days without symptoms Secondary: the duration of symptoms, the number of dates with little activity, and the usage of rescue medication	7-9 months
Perry TT et al. [24]	2018	USA	Cluster RCT	363	9.6 years	Asthma	44%	Consultations for asthma via telemedicine	Primary: number of days without symptoms Secondary: usage of peak flow meters, medication compliance, quality of life, self-efficacy, lung function, and understanding of asthma.	06 months
Erkkola-Anttinen N et al. [25]	2019	Finland	RCT	41	21 months	Otitis Media	42%	Otoscopy in home via telephone	Primary: ruling out otitis media Secondary: the effectiveness of instructional interventions and the diagnostic quality of the films	2 months
Coker TR et al. [26]	2019	USA	Cluster RCT	342	8.6 years	Mental Issues	38.3%	Video consultations for mental health issues	First: completing the screening visit Secondary: the duration between the referral, screening visit, and in-take visit	6 months

Use of Telemedicine Approaches

All of the included studies used a variety of telemedicine methods. Five studies (45%) [17, 18, 20, 23, 24] used conventional patient and physician visits via videoconferencing instead of in-person doctor appointments. Two studies (18%) [22, 25] involved parents completing tasks on their smartphones prior to the doctor's appointment, while three studies (27%) [21, 22, 25] employed smartphone-based telemedicine approaches. One activity required a parent to photograph a patient's skin condition in the medical facility's waiting area [24], and another involved a parent performing otoscopy at home [22]. Additionally, a blood glucose meter that synchronized patient data with an app that could alert doctors was used as a telemedicine strategy. Also, two studies primarily used telephone counseling [16, 19]; the first provided telephone dietary counseling, and the second included parent education on managing their children's behavior as well as nutrition counseling. In one trial, the control group communicated by phone, whereas the intervention group used telemedicine and videoconferencing. A screening visit was carried out using telemedicine in another study. Instead of a conventional in-person visit, a mental health clinic used videoconferencing for an initial examination [26].

Telemedicine and Asthma

A school-related telemedicine strategy was employed by Perry TT et al. [24] and Halterman JS et al. [23] to help patients manage their asthma symptoms. Halterman JS et al. found that the number of symptom-free days in the telemedicine group was significantly higher than in the control group (P=.01), whereas Perry TT et al. found no statistically significant difference between the total number of symptom-free days within the telemedicine and standard of care groups (P=.51) [24]. According to Perry TT et al., the telemedicine group significantly outperformed the conventional care group in terms of adherence to medication (P=.03) and peak flow sensor use (P<.001) [24]. Additionally, according to Halterman JS et al., a higher percentage of patients in the telemedicine category (181/199, 91%) received prescriptions for prophylactic medication, compared to the control group, the patients in the telemedicine group experienced lower hospitalization rates (14/199, 7%) [23]. Furthermore, compared to the control group, individuals in the telemedicine category experienced a substantially greater percentage of symptom-free days during the follow-up longitudinal visit (P<.02). At the conclusion of their research, Perry TT et al. and Halterman JS et al. found no discernible variations in the groups' quality-of-life scores [23, 24]. According to the Halterman JS et al. study, the majority of parents reported that the program was beneficial (361/377, 95.7%) and that they would participate in another one of a similar nature (365/377, 96.5%). Additionally, compared to families in the control group, families in the telemedicine group had a higher likelihood of learning more about medications for asthma (78.8%) [23].

Telemedicine and Obesity

Rhodes ET et al. [19], Fleischman A et al. [20], and Davis AM et al. [17] conducted specialized televisits, telephone dietary treatment, and doctor telemedicine measures, respectively, to examine the use of telemedicine in obesity management. Experts in obesity discovered that six months following the telemedicine intervention, the BMIs of all participants in the Fleischman A et al. study had dramatically dropped [20]. Rhodes ET et al. demonstrated that both groups' post-treatment levels of total calorie intake were significantly reduced by a low-glycemic index meal [19]. Additionally, the group on a low-glycemic load showed higher reductions in total energy consumption than the group on a low-fat diet. However, the two groups' changes in overall energy levels (that is, from the start of the therapy to the end of treatment) did not differ significantly. The BMI changes of parents and patients (i.e., from baseline to after treatment) did not significantly differ between the two groups, according to Davis AM et al. Participants in the telemedicine category found the service more beneficial than those in the control category [17], and the majority of patients (67%) in the Fleischman A et al. study said they preferred televisits to in-person specialist consultations [20]. Davis AM et al., on the other hand, found no discernible difference in the satisfaction ratings of the telephone and telemedicine categories [17]. Powers SW et al. studied how individuals with pancreatic insufficiency associated with cystic fibrosis responded to dietary counseling and instruction delivered via telehealth [16]. According to Powers SW et al., the control group's post-treatment energy consumption levels were much lower than those of the treatment group, and their height z-scores decreased more. There were no discernible variations in the two groups' post-treatment weight z-scores [16].

Telemedicine and Diabetes

Di Bartolo P et al. [21] used both the IBGStar blood sugar meter and a conventional blood glucose meter to measure variations in patients' blood sugar levels. This investigation revealed that HbA1c levels decreased in both groups. At the end of treatment, there were no appreciable variations in the two groups' HbA1c values. In the two groups, a similar proportion of patients self-monitored their blood glucose levels. Reductions in HbA1c levels were linked to self-monitoring of blood sugar levels. Compared to the group using the conventional meter, the telemedicine group reported higher reductions in HbA1c readings six months after therapy using the experimental IBGStar meter. Even a year after therapy, the HbA1c values in the experimental group remained constant. When comparing the two groups' quality-of-life metrics six and twelve months after therapy, no discernible differences were found. More calls were made to the doctor by individuals in the telemedicine category than by those in the control group.

Telemedicine and Mental Health

Coker TR et al. [26] investigated the effectiveness of in-person and telemedicine mental health assessment visits. Individuals in the telemedicine category needed more time to finish the screening visit, even though a higher percentage of them (80%) than those in the on-site group (64%), finished the visit. The number of patients who completed the in-person screening visit was not significantly impacted by the screening visit's method of delivery. Although there was no difference in the two groups' quality of life, individuals in the telemedicine category expressed greater satisfaction with the screening procedure than those in the in-person group.

Telemedicine and Parents Education

In two trials, Erkkola-Anttinen N et al. [25] and O'Connor DM et al. [22] required that parents acquire telemedicine skills to record their child's medical condition. Erkkola-Anttinen N et al. trained caregivers on how to perform an otoscopy of a patient's ear using a smartphone [25]. Parents were advised by O'Connor DM et al. to take a picture of their child's skin condition [22]. According to Erkkola-Anttinen N et al., recordings from parents who received smartphone otoscopy instructions (99%) were used to confirm or rule out acute otitis media diagnoses, and these diagnoses were more precise than those determined by videos from parents who weren't given instructions (58%) [25]. There was a noticeable difference in video quality between the instructional and non-instructed groups in the Erkkola-Anttinen N et al. study [25]. O'Connor DM et al., however, found no discernible difference between parents who received instruction and those that did not in terms of the concordance of in-person and photograph-based diagnoses [22]. Photographs that allowed for a diagnosis to be made had an average quality rating that was higher than that of photos that did not. According to Erkkola-Anttinen N et al., 56% of otoscopy videos with adequate diagnostic quality could be used to make a diagnosis [25]. In the O'Connor DM et al. study, the willingness of parents to employ teledermatology services was assessed on a scale of 1 to 10, with the median rating being 8 [22].

Discussion

According to the data in this review, telemedicine appointments for pediatric care could be as useful as in-person appointments. Eleven studies met all inclusion criteria. Each included study was a RCT that evaluated pediatric telemedicine use across eight medical conditions: otitis media, asthma, obesity, mental health issues, skin disorders, type 1 diabetes, attention deficit hyperactivity disorder (ADHD), and pancreatic insufficiency associated with cystic fibrosis. Instead of standard in-person doctor appointments, the majority of research employed videoconferencing visits. Additional telemedicine approaches included web-based screening appointments, telephone counseling, and smartphone applications.

Overall, telemedicine programs showed potential, even if their influence on pediatric healthcare was limited. Research on telemedicine therapies for asthma in schools has produced conflicting findings regarding how telemedicine affects days without symptoms of asthma [23, 24]. However, parents expressed satisfaction with the treatments and observed advances in outcome measures like medication adherence, asthma education, and the quantity of prescriptions for preventive medications. Similarly, patients stated that they either had no preference for in-person consultations or favored televisits over them, despite conflicting findings in research about the effect of telemedicine on weight loss. Additionally, patients expressed greater satisfaction with telemedicine methods than with in-person mental health screenings [26]. Furthermore, after participating in web-based educational and therapy sessions, parents' quality of life improved [18]. This implies that telemedicine services can enhance in-person encounters. Research has also shown that educating parents about telemedicine methods for monitoring and recording children with medical issues is a workable strategy that caregivers find acceptable [21, 25]. Furthermore, patients who used glucose monitoring devices according to telemedicine stated that they communicated with their doctors more often. This implies that digital methods for tracking long-term medical conditions can be enhanced by telemedicine technology [21].

Recent studies have proposed telemedicine techniques throughout pediatric practice as a way to improve access to healthcare, decrease inequalities, and offer alternatives to regular patient visits [27-29]. An objective of current studies has been to raise the bar for telemedicine providers so that they can offer better care at a lower cost. In pediatrics, telemedicine approaches have demonstrated potential in the care of chronic health conditions, particularly when combined with in-person approaches [30, 31].

Medicine has rapidly evolved in response to the recent SARS-CoV pandemic, with telemedicine emerging as a key delivery method. Research shows that telemedicine visits at critical and nonurgent care institutions have grown by 35% and 45%, respectively. Additionally, updates and improvements have added telehealth features to numerous pediatric patient portals [32]. However, it is important to note that not all telehealth responses have been equally efficient. For example, a study conducted in Georgia revealed significant discrepancies when comparing governmental telehealth responses to those of private telehealth clinics, with the latter showing more effective outcomes. Other studies have similarly pointed out variations in telehealth efficiency, emphasizing the need for improved standardization across different platforms and institutions [33]. These findings suggest that while telemedicine has expanded access, there are still challenges in ensuring consistent quality of care across different telehealth systems [34]. During the COVID-19 epidemic, telemedicine not only shields patients and healthcare professionals from unnecessary contact with sick patients, but it also saves protective clothing, which should be reserved for critical visits [35, 36]. Patients are being triaged and screened for COVID-19 symptoms using new telemedicine technology, such as bots that simulate conversations. However, given the growing use of telemedicine technology at clinics and hospitals, it is necessary to assess these technologies to understand how they affect insurance companies, employees, patients, and healthcare systems [37].

Giving children and their parents the best care possible, particularly during the COVID-19 epidemic, requires prompt management of juvenile chronic disorders like obesity, asthma, and genetic diseases [38]. Chronic illnesses and their associated medications have been managed with the use of web-based telemedicine consultations. Additionally, diabetes type 1 symptoms have been routinely recorded during the COVID-19 epidemic using blood sugar monitoring software [39]. Stress can exacerbate common symptoms like migraines, and telemedicine can help with treatment and reduce the need for hospital visits [40].

Additionally, telemedicine is being utilized in certain pediatric specialized settings. Telemedicine techniques in surgery have been applied to preoperative evaluations of patients, surgical procedures (such as using robotic devices), and postoperative patient monitoring [41]. Pediatric GI specialists have also monitored chronic illnesses (such as inflammatory bowel disease) and supplemented in-person appointments with telemedicine. Telemedicine referrals are also being utilized to maximize the availability of subspecialty resources because of the scarcity of pediatric specialist physicians in some areas [42]. According to a survey conducted at a pediatric migraine clinic in California, telemedicine appointments were easier for all participating families than in-person visits. Additionally, these families said they would choose to use telemedicine again. Telemedicine visits have attracted a lot of interest from families with children who have a wide range of medical concerns, and the majority of these families have access to adequate technology to make the visit [43].

Pediatric patients in remote areas face unique challenges, including lengthy travel to clinics and restricted access to subspecialty care [44]. However, telemedicine solutions can help overcome these obstacles. Pediatricians in rural parts of the US have supported telemedicine because it helps enhance patient connections and make specialized care more accessible. Patients (such as those from remote areas) can use telemedicine as a convenient platform to get the medical care they require, cut down on travel time, and shorten appointment wait times [45].

Telemedicine is used in pediatric and adult healthcare in comparable ways. Physicians in intensive care units can check the status of several patients at any time and from any location via web-based patient monitoring through telemedicine technologies [46]. In one study, web-based ECG and electroencephalogram devices were used to monitor neurology patients. Additionally, telemedicine tools can be used to shorten the time required to provide proper education and enhance preprocedural instructions [47].

Limitations

Despite setting the search strategies in a way that should include studies from all over the world, all of the included studies were conducted in high-income countries. The adoption of telemedicine may differ between high- and low-income countries, therefore the findings of this review should be interpreted as originating from countries with high incomes. All of the studies included were reported with different follow-up periods and populations of patients with different health conditions and gaps in the age groups. Therefore, there were inconsistencies in the results of each study and we were unable to run a meta-analysis.

## Conclusions

The application of telemedicine in Pediatrics has increased in recent years. Recent studies have demonstrated that telemedicine solutions are on par with or superior to in-person services, despite the lack of a clear consensus regarding the advantages of telemedicine techniques in pediatrics. Additionally, telemedicine sessions have consistently been rated as more satisfactory by patients and caregivers than in-person appointments. This demonstrates the potential of telemedicine in pediatric settings, particularly in situations like the COVID-19 pandemic where social isolation is necessary. Future research should concentrate on enhancing the efficacy of telemedicine approaches, integrating telemedicine with in-person doctor appointments, expanding access to healthcare, and improving telemedicine delivery systems. Furthermore, to fully assess the potential of telemedicine methods for enhancing the medical conditions of children and adolescents, more research is required that focuses on the cost-effectiveness of telemedicine, the application of telemedicine solutions in rural areas, and obstacles to the adoption of telemedicine technology.
